# Internal Hernia in a Liver Transplant Recipien: A Case Report

**DOI:** 10.1155/2013/923647

**Published:** 2013-09-12

**Authors:** Hironori Hayashi, Hiroyuki Takamura, Yoshinao Ohbatake, Masatoshi Shoji, Shin-ichi Nakanuma, Hisatoshi Nakagawara, Tomoharu Miyashita, Hidehiro Tajima, Hirohisa Kitagawa, Takashi Tani, Koichi Shimizu, Tetsuo Ohta

**Affiliations:** ^1^Department of Gastroenterologic Surgery, Division of Cancer Medicine, Graduate School of Medical Science, Kanazawa University, 13-1 Takara-machi, Kanazawa, Ishikawa 920-8641, Japan; ^2^Department of Surgery, Public Central Hospital of Matto Ishikawa, 3-8, Kuramitsu, Hakusan, Ishikawa 924-0865, Japan; ^3^Department of Surgery, Toyama Prefectural Central Hospital, 2-2-78 Nishinagae, Toyama, Toyama 930-8550, Japan

## Abstract

Biliary complications have great importance for liver transplant recipients because of affecting long-term prognosis. In rare situations, an internal hernia of the Roux-en-Y loop cause graft injury. A 42-year-old woman with a history of living donor liver transplantation 6 years ago presented with prolonged graft injury during the past 6 months. She suddenly developed ileus of the small bowel with internal hernia through the defect of the mesentery around the Roux-en-Y limb of the hepaticojejunostomy. Emergent surgery was performed to reduce the hernia and volvulus; also the mesenteric rent was closed with interrupted suture of silk. Internal hernia of the small bowel after liver transplantation is rare but causes graft injury due to associated biliary complications and rapid deterioration of patient's condition.

## 1. Introduction

Liver transplantation has become an accepted therapeutic procedure for patients with end-stage liver disease, and its outcome has become satisfactory [1, 2]. However, some liver recipients have experienced graft loss due to postoperative morbidities, such as infection or rejection. In particular, biliary complications are very important because they affect long-term prognosis [3]. In rare situations, an internal hernia of the Roux-en-Y loop has become the cause of cholestasis. Here, we report a patient with internal hernia of the small bowel after hepaticojejunostomy performed during living donor liver transplantation (LDLT).

## 2. Case Report

A 42-year-old woman presented to her local emergency room with symptoms of episodic nausea and sudden abdominal pain. She had undergone living donor liver transplantation with biliary reconstruction as hepaticojejunostomy for primary sclerosing cholangitis at the age of 36 year, and was under tacrolimus immunosuppression. During the past 6 months, her ductal enzyme levels were significantly elevated, and cholestasis was suspected. She was admitted to our institute a few weeks prior to this episode for close examination. During the hospitalization, she needed to undergo percutaneous liver biopsy and transhepatic biliary drainage. The drainage exudates looked like diluted bile, and the amount was about 1000 mL/day. The biopsy specimen revealed chronic cholestasis with fibrosis. Signs of chronic rejection were not detected in the specimen.

**Figure 1:**
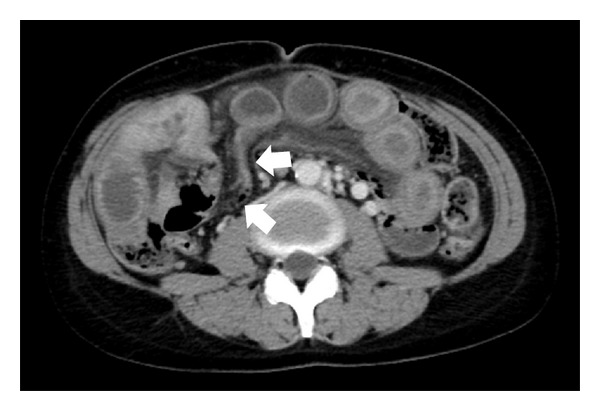
Abdominal computed tomography before surgery. Computed tomography demonstrated internal hernia of the small intestine (arrow: small intestine through the hernia orifice).

Abdominal computed tomography (CT) images revealed ascites and markedly dilated small bowel loops filled with small intestinal juice. Additionally, the small intestine was evident through the orifice of the internal hernia (Figure 1). These findings suggested ileus of the small bowel with internal hernia and volvulus. Thus, the patient was referred to our department for surgical treatment.

At the time of admission, she had diffuse abdominal guarding and rigidity with increased bowel sounds. Her laboratory data revealed leukocytosis and liver injury; therefore, she underwent emergent laparotomy.

**Figure 2:**
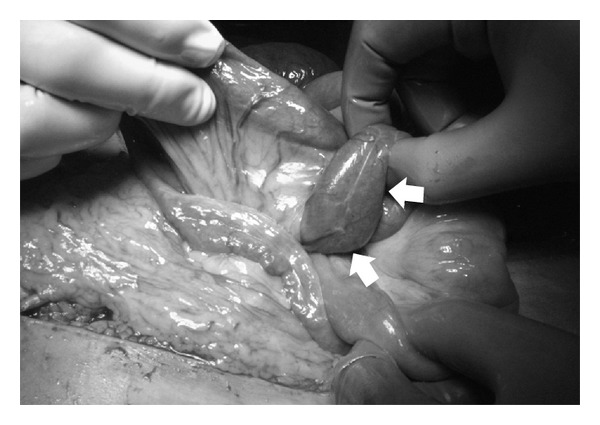
Photograph of operative findings. Internal hernia was evident through the rent of the mesentery around the Roux-en-Y limb of the hepaticojejunostomy (arrow: small intestine through the hernia orifice).

During the operation, the patient was found to have internal herniation and volvulus of the small bowel through the defect of the mesentery around the Roux-en-Y limb of the hepaticojejunostomy (Figure 2). The hernia and resulting volvulus were reduced, and the bowel was found to be viable. The mesenteric rent was closed with interrupted suture of silk. Postoperatively, the patient made a good recovery. The graft injury was reduced, and the patient is alive and well 2 years after surgery.

## 3. Discussion

Liver transplantation has become an accepted therapeutic procedure for patients with end-stage liver disease, and its outcome has become satisfactory [1, 2]. Following liver transplantation, biliary complications and their etiologies are very important because they affect graft function and long-term prognosis [3]. And many reports according to biliary complication, its prevention, and therapy were presented [4, 5]. Thus, rare causes of biliary complications, such as internal hernia of the Roux-en-Y loop in the present case, should be kept in mind.

There have been detailed reports of 11 cases (including the current case) of internal hernia related to the hepaticojejunostomy after liver transplantation (Table 1) [6–9]. In the reports, the orifice of the internal hernia was classified as follows: around the Roux-en-Y loop: 7 cases; mesenteric window: 4 cases.

Among the 11 cases, 4 patients received a right lobe graft, and 2 patients received a left lobe graft. In other 5 patients, graft type was not specified in the reports. The time of onset of internal hernia after liver transplantation varied from 13 days to 11 years. Computed tomography was performed in 8 patients, and the correct diagnosis was obtained in 7 patients. In spite of emergent surgery, 1 patient died. Thus, in patients having abdominal pain and a history of liver transplantation with hepaticojejunostomy, emergent CT is important for prompt and correct diagnosis.

Once an internal hernia without strangulation develops, cholestasis can occur occasionally due to outflow obstruction of the efferent loop. Stenosis of the hepaticojejunostomy and intrahepatic bile duct can result from the inflammatory change caused by the repetitive cholangitis [9]. Elevation of the ductal enzyme levels and graft injury can also occur as a result of these pathological reactions. In the present case, the etiology of the graft injury was thought to be as mentioned above. In addition, the patient was suspected to have had the internal hernia for a long time.

In order to prevent internal hernia in transplant recipients, the most important and essential technical point is to close the defects with nonabsorbable suture materials [6]. Absorbable suture materials do not have appropriate sustainability due to their potential inflammatory response under standard immunosuppressive therapy (calcineurin inhibitors and corticosteroids). Thus, mesenteric defects closed with absorbable sutures may fall apart.

## 4. Conclusion

For liver transplantation recipients, stasis of enteric fluid due to internal hernia can cause biliary complications and graft injury. This complication might be rare but can be avoided by careful mesenteric defect closure with nonabsorbable suture materials when performing Roux-en-Y hepaticojejunostomy or duct-to-duct biliary reconstruction.

## Figures and Tables

**Table 1:** Summary of the clinical data of all reported cases.

Author/year	Age/sex	Graft	Porte of hernia	Onset of hernia after LTx.	Diagnosis by	Therapy	Prognosis
Newton et al./1988 [7]	29 y.o./F	N. A.	Around Roux-en-Y	N. A.	Laparotomy	Repair of hernia without BR	Alive
Khanna et al./1997 [8]	12 y.o./F	N. A.	Around Roux-en-Y	10 years	CT	Repair of hernia, BR and small bowel transplantation	Alive
Khanna et al./1997 [8]	14 y.o./F	N. A.	Mesenteric window	2 years	Laparotomy	Repair of hernia without BR	Alive
Khanna et al./1997 [8]	12 y.o./F	N. A.	Mesenteric window	13 days	Laparotomy	Repair of hernia and with BR	Dead
Khanna et al./1997 [8]	38 y.o./F	N. A.	Mesenteric window	19 months	Laparotomy	Repair of hernia without BR	Alive
Liu et al./2004 [6]	56 y.o./M	Right lobe	Around Roux-en-Y	20 months	CT	Repair of hernia without BR	Alive
Liu et al./2004 [6]	50 y.o./M	Right lobe	Around Roux-en-Y	19 months	CT	Repair of hernia without BR	Alive
Liu et al./2004 [6]	44 y.o./F	Right lobe	Mesenteric window	23 months	CT	Repair of hernia without BR	Alive
Liu et al./2004 [6]	41 y.o./M	Right lobe	Around Roux-en-Y	17 months	CT	Repair of hernia without BR	Alive
Eberhardt et al./2012 [9]	12 y.o./M	Left lobe	Around Roux-en-Y	11 years	CT	Repair of hernia without BR	Alive
Our case/2013	42 y.o./F	Left lobe	Around Roux-en-Y	6 years	CT	Repair of hernia without BR	Alive

LTx: liver transplantation; N. A.: not available; CT: computed tomography; BR: bowel resection.
